# Complete remission from peritoneal metastasis of late recurrent breast cancer by endocrine therapy: a case report

**DOI:** 10.1186/s40792-020-01002-9

**Published:** 2020-12-09

**Authors:** Azusa Fuke, Isao Tabei, Tomoyoshi Okamoto, Hiroshi Takeyama

**Affiliations:** 1 Department of Surgery, Daisan Hospital, The Jikei University School of Medicine, 4-11-1 Izumihoncho, Komae-shi, Tokyo 201-8601 Japan; 2 Department of Surgery, Daisan Hospital, The Jikei University School of Medicine, 4-11-2 Izumihoncho, Komae-shi, Tokyo 201-8601 Japan; 3 Department of Surgery, Daisan Hospital, The Jikei University School of Medicine, 4-11-3 Izumihoncho, Komae-shi, Tokyo 201-8601 Japan; 4 Division of Breast & Endocrine Surgery, Department of Surgery, The Jikei University School of Medicine, 3-19-18 Nishishimbashi, Minato-ku, Tokyo, 105-8471 Japan

**Keywords:** Breast cancer, Late-phase metachronous peritoneal dissemination, Endocrine therapy

## Abstract

**Background:**

Peritoneal dissemination associated with the postoperative recurrence of breast cancer is relatively low (3–6%). Although the prognosis of patients with peritoneal metastasis is generally short (7–26 months), we experienced a unique case in which complete remission was achieved for more than 6 years with endocrine therapy alone.

**Case presentation:**

An 81-year-old woman presented an upper abdominal tumor and loss of appetite. Computer tomography (CT) scan revealed a tumor in the duodenum and the head of pancreas, which malignant lymphoma was suspected. The exploratory laparotomy demonstrated a tumor located in the greater curvature of the pylorus to the transverse colon, and peritoneal dissemination. Because of the previous history of breast cancer 11 years ago and the immunopathological findings, recurrence of breast cancer was diagnosed. Lung metastasis was also detected postoperatively and the endocrine therapy using letrozole was introduced. After a year, CT scan confirmed complete remission from the metastasis. Two years later, tumor markers fell within the normal limit.

**Conclusions:**

A rare case of late-phase metachronous peritoneal metastasis of the breast cancer where complete remission was obtained by a single endocrine agent was presented.

## Background

Peritoneal metastasis of breast cancer is relatively rare (3–6%) [1, 2], and the prognosis is generally short (7–26 months) [3]. In this paper, we report a case of peritoneal metastasis from breast cancer appearing 11 years after operation. Chemotherapy and/or endocrine therapy combined with molecular targeted drugs was considered the practical choice and endocrine therapy is recommended for elderly patients by various therapeutic guidelines [4–6]. Single agent endocrine therapy was chosen considering the background and the age of the patient and showed complete remission. We present this case along with the relevant literature review.

## Case presentation

**Fig. 1:**
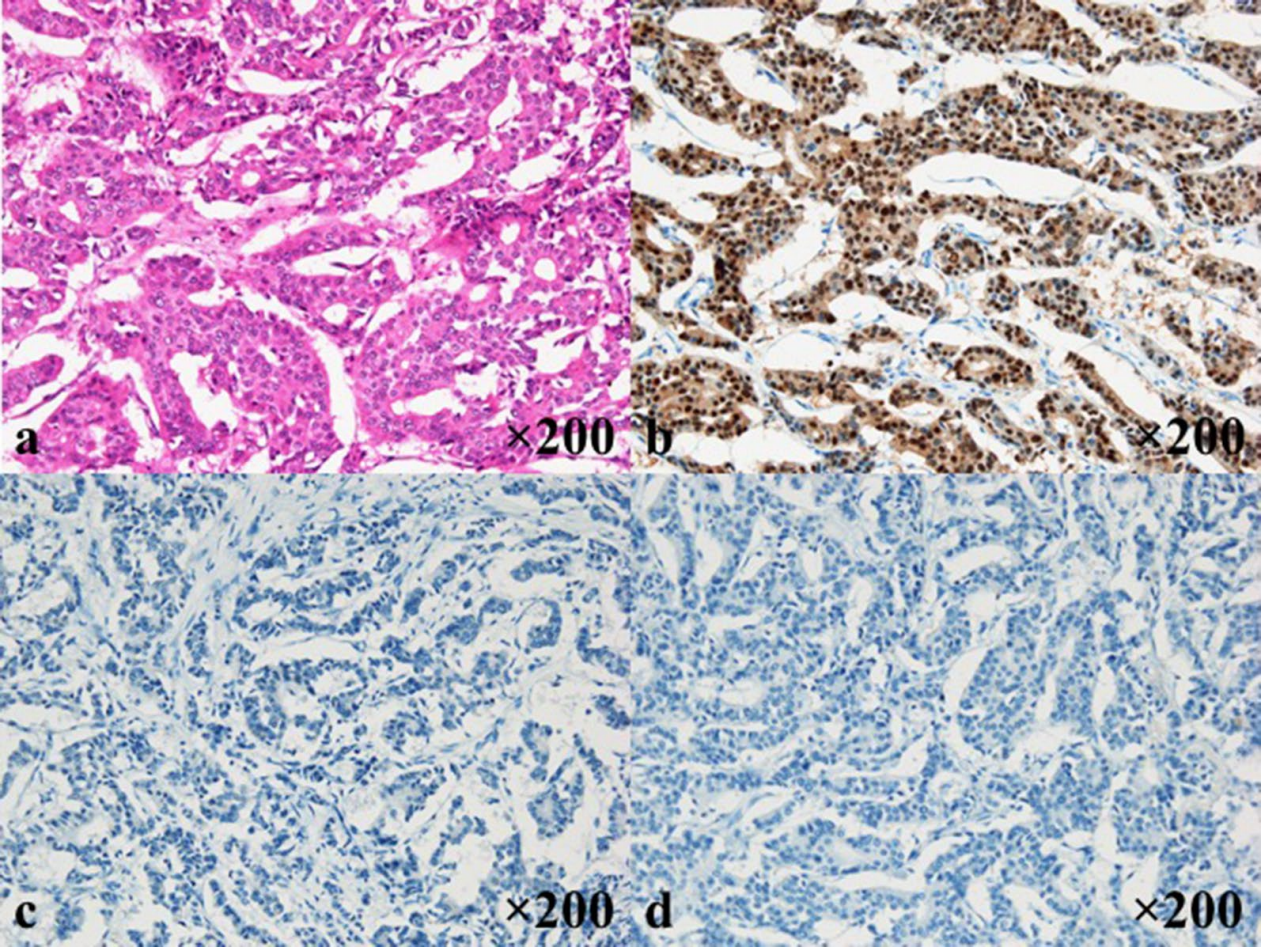
Pathological findings of the primary breast cancer. Pathological sections were cut and stained at the time of relapse, due to no information available from the original episode. **a** H&E staining (×200), **b** ER-positive staining of 80% (×200), **c** PgR-negative (×200), **d** HER2 receptor of 0 (×200)

**Fig. 2:**
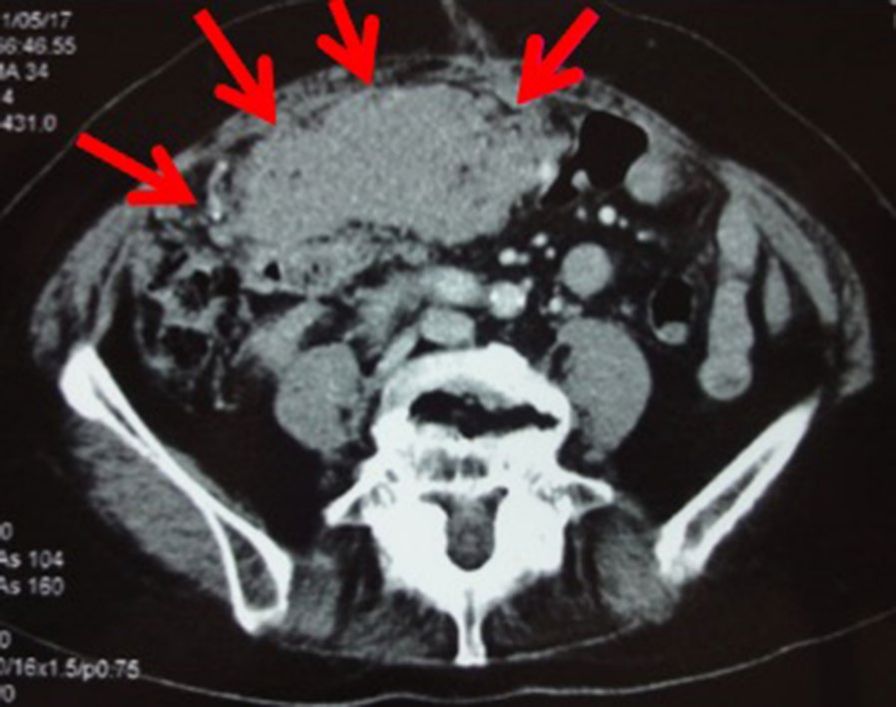
Preoperative findings by abdominal CT scan. A tumor about the size of one's palm was observed in the duodenum and the head of pancreas. The tumor was compressing the duodenum, causing loss of appetite and detention. Arrows (→) show the tumor’s margin occupying the abdominal cavity

**Fig. 3:**
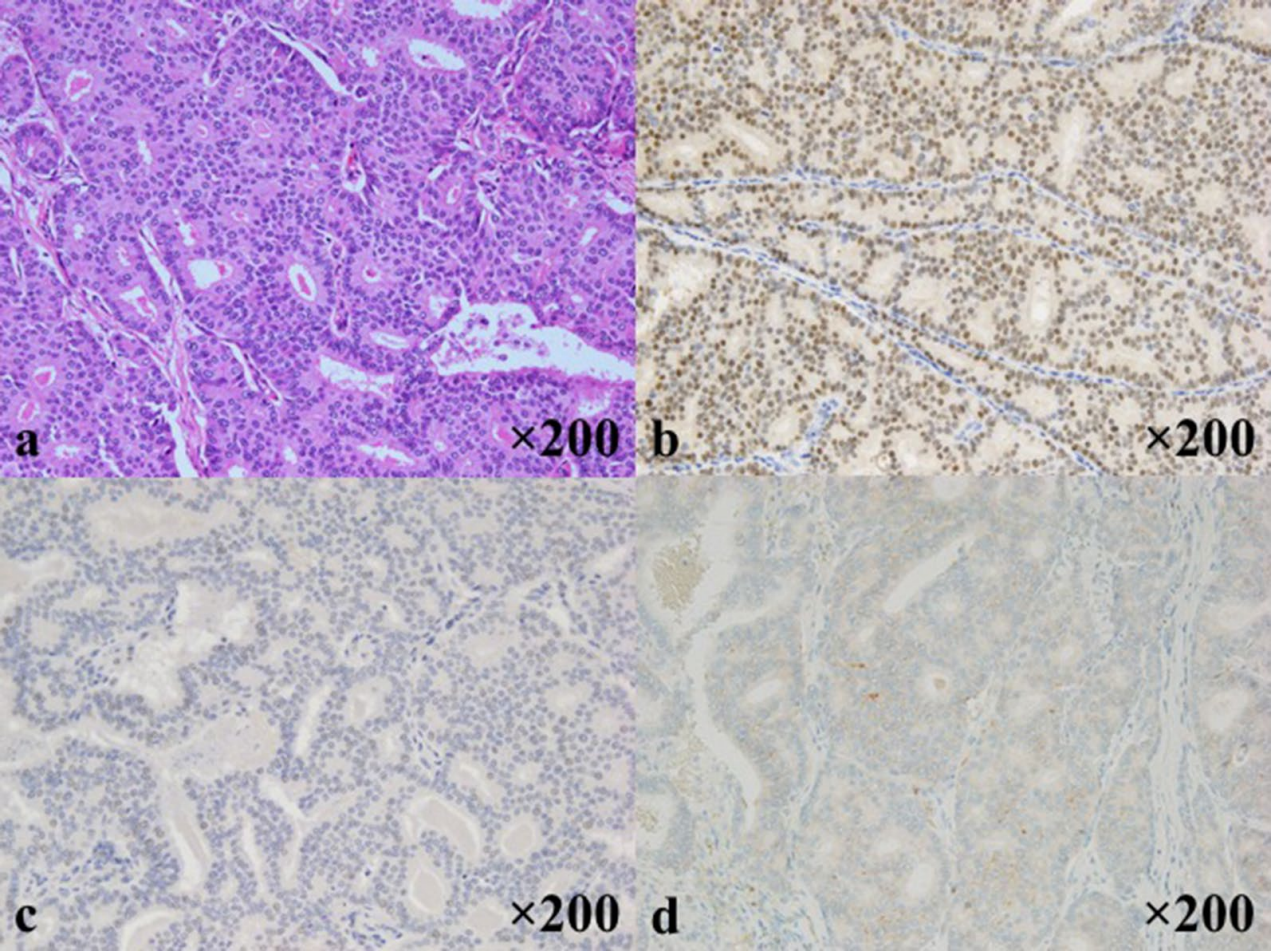
Pathological findings of the metastatic lesion. Characteristics resemble the primary breast cancer of 11 years ago. Pathological diagnosis was metastatic ductal carcinoma. **a** H&E staining (×200), **b** ER-positive staining of 70% (×200), **c** PgR-positive staining of 5% (×200), **d** HER2 receptor of 1+ (×200)

**Fig. 4:**
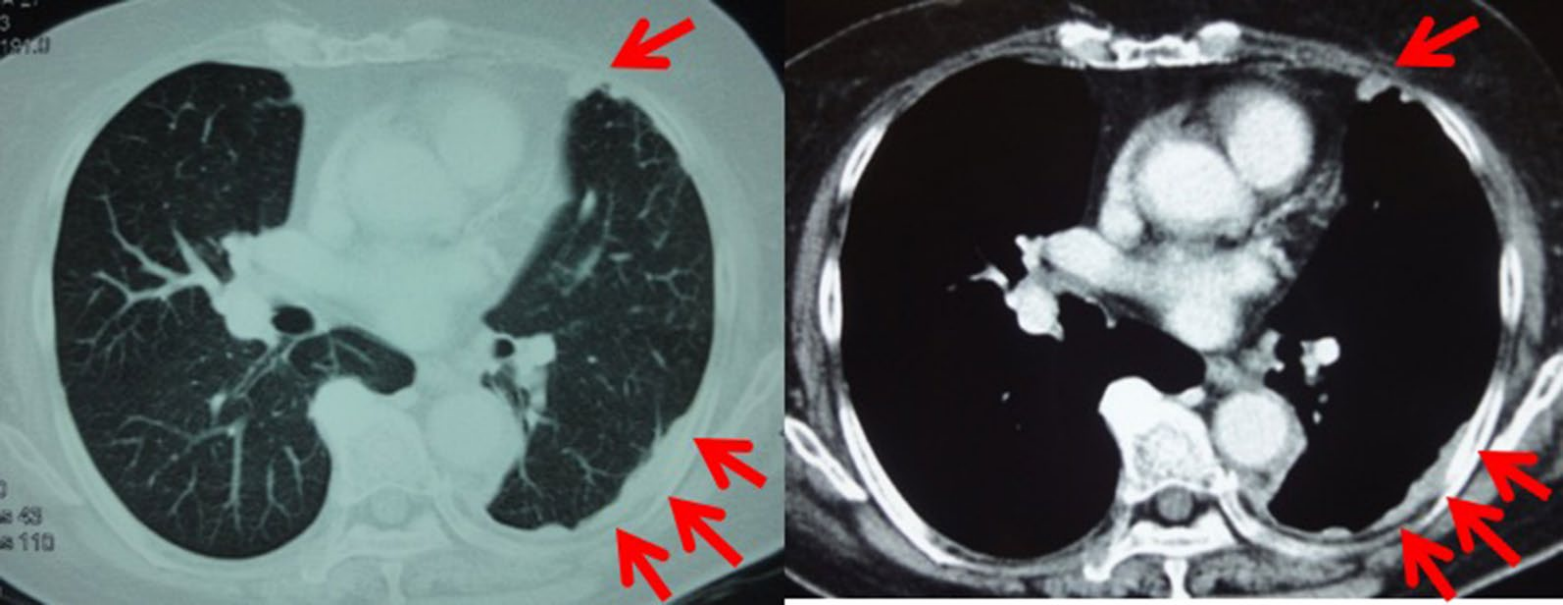
Pleural metastasis was observed in the postoperative chest CT scan. Arrows (→) show pleural metastasis and pleural effusion

An 81 year-old-woman visited the department of internal medicine in our hospital complaining of an upper abdominal mass, loss of appetite, and abdominal distention. An elastic and hard tumor was palpated in the right hypochondriac region. Other than hypertension, she had a history of left breast cancer 11 years ago. She had no family history of breast cancer. Left breast-conserving operation therapy along with axillary lymph node dissection and postoperative radiation therapy were performed in May 2000. Histopathological examination revealed invasive ductal carcinoma, including papillotubular carcinoma with pT2N0M0 (pStageIIA). Specific immunostaining revealed results of estrogen receptor (ER)-positive staining of 80%, progesterone receptor (PgR)-negative, human epithelial growth factor receptor 2 (HER2) of 0. These stainings were done at the time of relapse (Fig. 1). The endocrine therapy was not performed because the patient did not select it. Ultrasonography and CT scan revealed a tumor about 16 × 10 cm in the duodenum and the head of pancreas compressing the duodenum. Malignant lymphoma in particular was suspected based on the CT image (Fig. 2). Diagnostic biopsy to the surgical department was requested and an exploratory laparotomy was performed. No abnormal values were obtained in the blood biochemical test. Serum tumor marker test concerning breast cancer was not performed preoperatively. Operation under general anesthesia was performed to retrieve biopsy samples and explore the inner abdomen. Among examination of the abdominal cavity, nodules and relatively large tumors were scattered all over the peritoneum. The largest tumors were mainly observed in the greater omentum. The tumors had unevenly raised surfaces and covered a large area from the greater curvature of the pylorus to the transverse colon. A 40 × 30 mm tumor was resected. The sample was diagnosed as an adenocarcinoma by intraoperative rapid pathological diagnosis. The operational diagnosis was peritoneal dissemination of a cancer of an uncertain origin rather than malignant lymphoma. Ductal carcinoma was suspected due to the following histopathological analysis of the sample performed postoperatively (Fig. 3). Specific immunostaining resulted in ER-positive staining of 70%, PgR-positive staining of 5%, HER2 of 1+, Ki-67-positive staining of 5%. In consideration of the patient’s history and intra-abdominal search through radiological images, surgical exploration, and pathological finding, it was diagnosed as peritoneal metastasis of recurrence breast cancer. All other candidates for the primary sites, including the ovary were ruled out. A whole-body evaluation was performed postoperatively, and pleural metastasis and pleural effusion were observed in the chest CT scan (Fig. 4). Other local recurrences, or distant recurrences in bone or brain were not observed. Tumor marker results showed normal CEA values (4.4 ng/ml), but elevated values for BCA225 (4300 U/ml), CA15-3 (214 U/ml), and NCC-ST-439 (7.4 U/ml) were obtained. In consideration of breast cancer subtype (luminal A like) and the patient age (81 years), the endocrine therapy using letrozole was introduced.

**Fig. 5:**
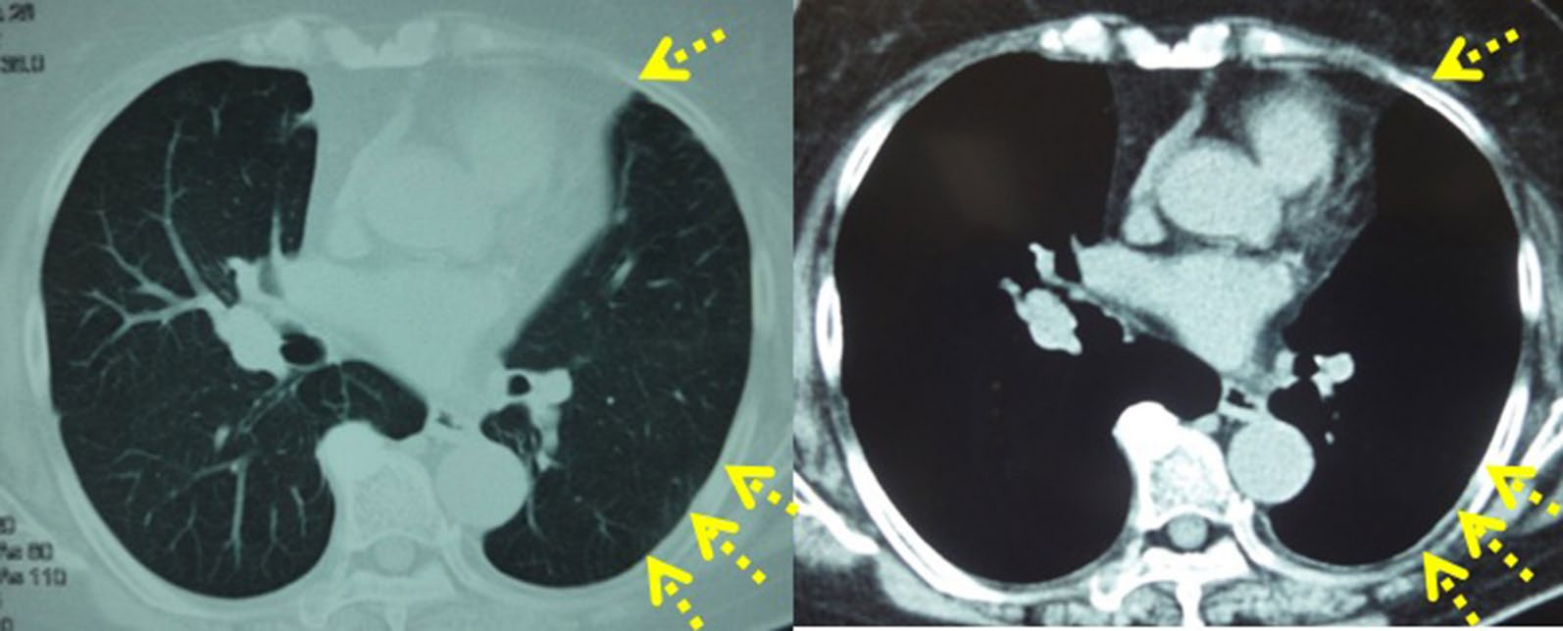
Complete recovery from pleural metastasis could be confirmed by a chest CT scan performed 1 year after endocrine therapy was started. Arrows (→) show disappearance of pleural metastasis and pleural effusion

**Fig. 6:**
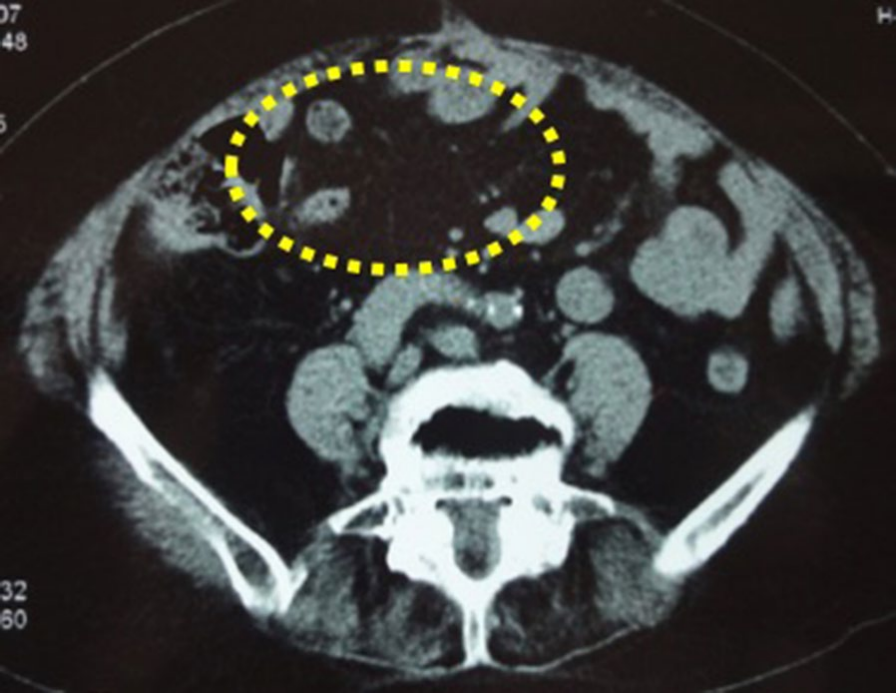
Complete recovery from intra-abdominal metastasis could be confirmed by an abdominal CT scan performed 1 year after endocrine therapy was started. Spotted circle (…) show the disappearance of intra-abdominal metastasis

**Fig. 7:**
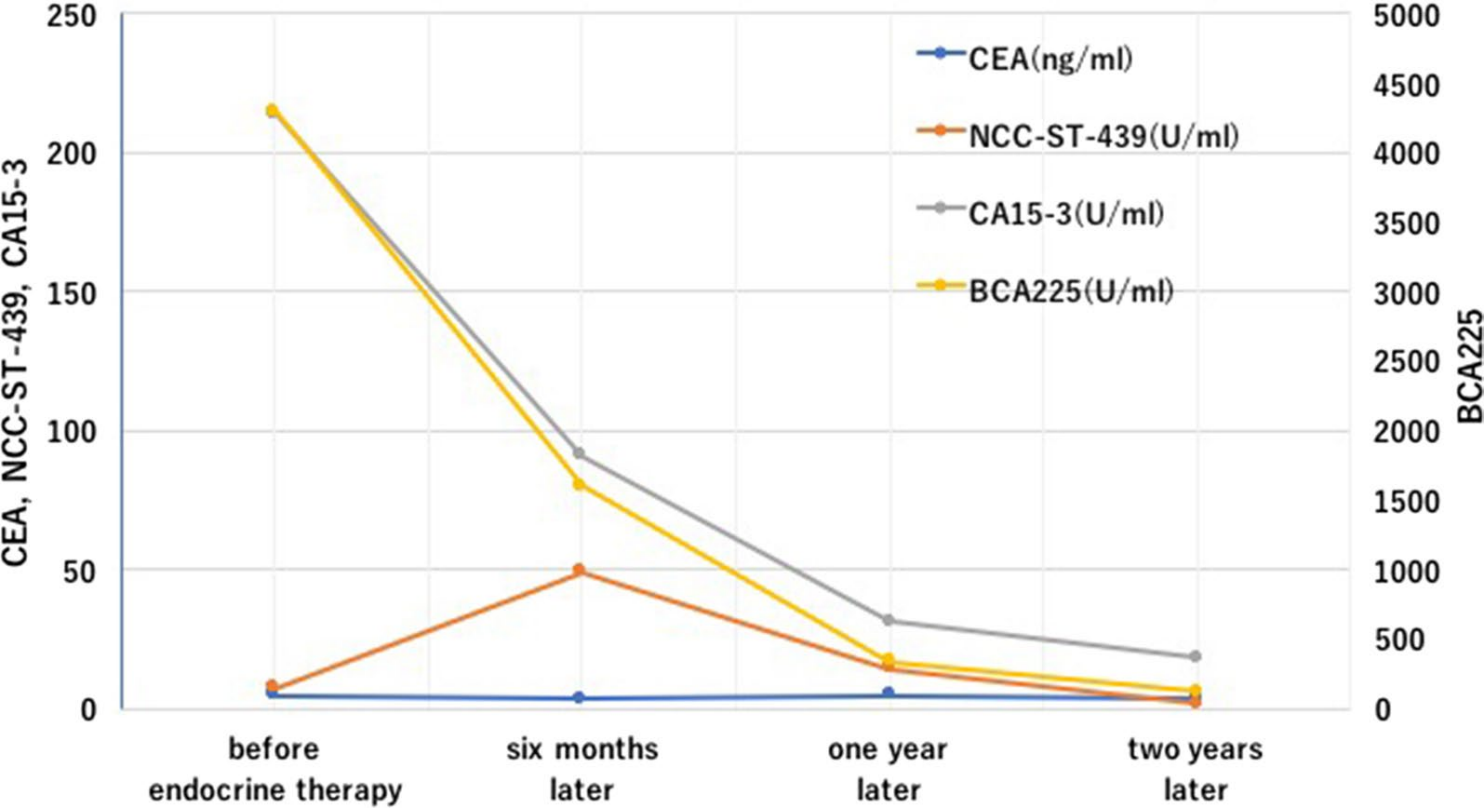
Variations in the tumor marker values. No abnormal CEA values were obtained. CA15-3 and BCA225 values gradually declined from the time of recurrence, and returned to normal levels 2 years later. NCC-ST-439 values gradually increased up to 49 U/ml 6 months after relapse, and gradually decreased thereafter, reaching normal levels 2 years later

Course of treatment with 2.5 mg of daily letrozole was prescribed to the patient. In the first year of endocrine therapy, recovery from pleural metastasis and pleural effusion could be confirmed by chest CT scan (Fig. 5). Abdominal CT scan also showed reduction in the intra-abdominal metastasis (Fig. 6). Regarding tumor markers, no abnormal CEA value was obtained. CA15-3 and BCA225 values gradually declined from the time of recurrence and returned to normal levels 2 years later. NCC-ST-439 value gradually increased up to 49 U/ml 6 months later, with a gradual decline there after along with the other tumor markers (Fig. 7). The patient was currently receiving treatment in the outpatient. Six years have passed, and no relapse of the malignancy was evident and the patient is still alive on the same medication.

## Discussion

Breast cancer is known to show metastasis highly to liver, bone, lung, pleura, and soft tissue. Metastasis to the gastrointestinal tract or the peritoneum is relatively rare. Regarding correlation between histological type and metastasis site, solid-tubular carcinoma is reported to be highly metastatic to liver and bone, and papillotubular carcinoma and scirrhous carcinoma to the peritoneum [7]. Also, peritoneal dissemination was reported to be more frequent in a case of invasive lobular carcinoma than in a case of invasive ductal carcinoma [8].

Tabei et al. [9] reported that the incidence rate of peritoneal metastasis from advanced and recurrent breast cancer is 3.0%. Mukaiyama et al. [10] reported that the diagnostic rate of metastasis in the gastrointestinal tract or the peritoneum is 6% in living patients, and 31% in autopsy specimens. The higher rate obtained in autopsy specimens maybe due to the fact that candidates for autopsy would be the patients that experienced peritoneal dissemination and metastasis to the gastrointestinal which is often lethal to the patient. Also, it may be the reason why during follow-up of postoperative breast cancer, abdominal examination may not always be highlighted. In terminal clinical condition, in addition to peritoneal metastasis, metastasis to other parenchymal organs, such as lymph nodes, along with lung and liver, were also reported. Since metachronous (late stage) metastasis is more common than synchronous metastases [11, 12], diagnosis of the disease at the first onset is difficult to be made before the operation of the breast cancer [13]. As is with this case, the patient visited the hospital 11 years after the original breast cancer surgery complaining for loss of appetite and a feeling of abdominal distention. In the preoperative imaging test, malignant lymphoma was suspected rather than recurrent breast cancer, due to the long absence of the original disease. The prior preoperative diagnosis made by the attending doctor of internal medicine was malignant lymphoma from the clinical symptoms in need of exploratory laparotomy for final diagnosis. Peritoneal metastasis from the breast cancer could be diagnosed only by the pathological results obtained by the resected sample at the exploratory laparotomy.

Since the patient had loss of appetite and compression of the duodenum was found in the preoperative CT scan, ileus was expected to occur sooner or later. An exploratory laparotomy was first performed for diagnosis. As a result, peritoneal dissemination due to metastasis of breast cancer was diagnosed. Although in additive from the postoperational examinations metastases in vital organs, namely pleural metastasis, were observed, endocrine therapy was selected, which was the primary choice used for the treatment of luminal A like type breast cancer [14]. A single agent oral aromatase inhibitor, letrozole was chosen, considering the background and age of the patient. Chemotherapy was also a choice for vital organ relapse of breast cancer patients, but the adverse effects were determined to be lethal for this particular patient. Recently, endocrine therapy along with the addition of cyclin-dependent kinase4/6(CDK4/6) inhibitors have been reported very promising results [15]. This agent would definitely be a candidate, but the availability was still not possible at the time. Still the chosen single agent endocrine therapy was successful, and after 2 years of continuous treatment, findings of metastasis were not observed by imaging and values of tumor marker were normalized. Although more than 6 years have passed since the onset of recurrence, no evidence of further recurrence has been observed. The patient is no longer receiving active therapy against metastatic breast cancer due to onset of dementia, but her survival was accounted for more than 8 years after the metastatic recurrence of peritoneal dissemination of the breast cancer.

McLemore et al. [3] reported that the median overall survival time of patients with peritoneal metastasis was 14–26 months, while a median survival period of 7 months has been reported by Chu et al. [1]. On the other hand, the average life expectancy was approx. 18 months, and the 5-year survival rate was 13% in patients with stage IV breast cancer [16]. Prognosis for peritoneal metastasis is considered lower than other cases of stage IV breast cancer. In the recent years, however, long-term survival cases have also been reported [17–19].

Recently, there are some reports on treatments which have achieved long-term survival in patients with peritoneal carcinomatosis of breast cancer, but detailed treatment outcomes have not been reported. For this reason, a standard therapy has not been established. However, the appropriate treatment for breast cancer as the primary disease is basically applied, namely, multidisciplinary therapy (operation, chemotherapy, endocrine therapy, and molecular targeted drug) [20].

Recent developments in diagnostic technology and treatment methods, such as chemotherapy have achieved a drastic improvement in the prognosis of breast cancer. Hence, along with an improved survival rate of breast cancer, the number of cases with late recurrent carcinomatous peritonitis, even after 11 years as in this case, may also increase. Late recurrence of peritoneal metastasis should be considered when diagnosing patients with a history of breast cancer. There are few reports on peritoneal metastasis from breast cancer, and no standard therapy has been established. Further accumulation of clinical cases and consideration of effective treatment based on these cases are expected.

## Conclusions

We experienced a unique case of peritoneal dissemination and metastasis from breast cancer which complete remission by endocrine therapy was achieved.

## Data Availability

There is no available data and materials to be shared.

## Abbreviations

CT: Computer tomography
ER: Estrogen receptor
PgR: Progesterone receptor
HER2: Human epithelial growth factor receptor 2
CDK4/6: Cyclin-dependent kinase4/6
